# The Large Scale Synthesis of Aligned Plate Nanostructures

**DOI:** 10.1038/srep29972

**Published:** 2016-07-21

**Authors:** Yang Zhou, Philip Nash, Tian Liu, Naiqin Zhao, Shengli Zhu

**Affiliations:** 1 Thermal Processing Technology Center, Illinois Institute of Technology, Chicago, IL 60616, USA; 2 Key Laboratory of Composite and Functional Materials, School of Materials Engineering, Tianjin University, Tianjin, 300072, China

## Abstract

We propose a novel technique for the large-scale synthesis of aligned-plate nanostructures that are self-assembled and self-supporting. The synthesis technique involves developing nanoscale two-phase microstructures through discontinuous precipitation followed by selective etching to remove one of the phases. The method may be applied to any alloy system in which the discontinuous precipitation transformation goes to completion. The resulting structure may have many applications in catalysis, filtering and thermal management depending on the phase selection and added functionality through chemical reaction with the retained phase. The synthesis technique is demonstrated using the discontinuous precipitation of a γ′ phase, (Ni, Co)_3_Al, followed by selective dissolution of the γ matrix phase. The production of the nanostructure requires heat treatments on the order of minutes and can be performed on a large scale making this synthesis technique of great economic potential.

Nanostructured materials have great potential in a large number of applications that can benefit society^1^. Many applications such as catalysis and filtering take advantage of the high surface area and porosity that can result from nanostructures^2^,^3^. However the synthesis of nanoscale materials often involves the use of chemical techniques that are inefficient and difficult to scale up^4^ or are simply too slow to apply in large-scale manufacture. An additional problem is how to arrange the nanomaterial so that it can be effectively utilized since it is often in the form of discrete particles or plates^5^,^6^. Experience has shown that issues of agglomeration of the nanomaterial and adherence to a substrate arise that often requires compromising the functionality of the material. It is therefore beneficial to develop synthesis techniques to produce self-assembled, self-supported nanostructures on a large scale.

Precipitation from a solid solution produces discrete second-phase particles, whose size can be controlled through alloy composition and thermal processing. In metallic systems precipitation of a second phase may also occur in a discontinuous manner producing plates or rods of the second phase^7^,^8^,^9^. This latter process, known as discontinuous or cellular precipitation (DP), can lead to plates with a distinct orientation relationship with the matrix and nanoscale widths and spacings^9^. The DP transformation nucleates at grain boundaries and grows by motion of the grain boundary, as shown in Supplementary Fig. S1. Thus the process is controlled by grain boundary diffusion rather than volume diffusion as in the case of continuous precipitation. The process to produce a complete DP transformation can be of short duration and there is potential for the large scale synthesis of aligned plate nanostructures. In alloy systems in which the DP transformation goes to completion, one could selectively dissolve one of the phases to produce a material with aligned^10^,^11^, self-assembled nanostructured plates with nanoscale spacings between them, as shown in Fig. 1. In many cases DP transformations do not go to completion due to a decreasing driving force resulting from continuous precipitation (CP) ahead of the advancing DP interface^12^. This competition between the two modes of precipitation often produces only a small volume fraction of DP. For structural alloys this is an undesirable microstructure since it is detrimental to the mechanical properties and as a result the usefulness of the DP transformation has been neglected, although the potential value of a complete DP transformation was pointed out several decades ago^13^.

In earlier reports, it has been shown that discontinuous precipitation in the Ni-Co-Al alloy system can go to completion at low aging temperatures and at high Co contents^9^. Figure 2 shows the isothermal section of the Al-Co-Ni system at 800 K^14^. The reason for the increase in propensity for discontinuous precipitation with increasing cobalt content has not been established. One hypothesis is that the effect of an increasing requirement for partitioning of the slower diffusing element, cobalt, as the cobalt content increases (indicated by the rotation of the tie lines, see Fig. 2), favors a grain boundary diffusion process over a volume diffusion process. Figure 2 shows that at low Co contents the two phases contain almost the same amount of Co (tie lines parallel to the Ni-Al side). As the Cobalt content increases, there is a rotation of the tie line so that at the limit of the 2 phase field bordering the γ + γ′+ β phase field there is 35 at.% Co in Ni_3_Al (γ′) and 86 at.% Co in the Ni solid solution (γ) phase with which it is in equilibrium. Another possibility for the increased propensity for DP is a change in the γ/γ′ lattice mismatch causing a change in the interfacial energy favoring discontinuous over continuous precipitation^9^. Further fundamental understanding of the kinetics of the DP transformation is needed so that appropriate heat treatment parameters may be selected for other alloy systems that have potential for complete DP transformations.

Previous work to produce self-assembled nanostructured γ′ precipitate architectures in a Ni-base alloy through selective dissolution resulted in processes involving application of stress to a material at high temperature over a period of hundreds of hours^15^,^16^,^17^. Such approaches are impractical from both a productivity and economic perspective.

This work is directed at addressing issues of self-assembly, structural alignment and scalability for production of nanoscale plates through a novel synthesis technique, which involves transforming a solid solution by discontinuous precipitation followed by selective etching to remove one of the phases. A schematic process for the production of the nanoscale architectures is shown in Fig. 3. The technique is generally applicable to any alloy system undergoing discontinuous precipitation by a coupled growth mechanism resulting in an aligned two-phase structure, in which one phase can be removed by selective dissolution.

## Results

### Metallography

The alloy composition selected to demonstrate this synthesis technique was Ni-49at.%Co-12at.%Al, henceforth referred to as Ni49-12. The development of the discontinuous precipitation transformation as a function of aging time and temperature was investigated using optical microscopy. The starting grain size after solution treatment was 34 μm. Figure 4 shows the optical microstructures of the alloys after aging at 600 °C. It can be seen in Fig. 4a that after aging for 2 min, the amount of DP transformation (dark regions in the micrograph) volume fraction was around 25%. After 5 min at 600 °C, nearly 85% transformation is achieved (Fig. 4b) and with increase of annealing time to 30 min, the discontinuous precipitation transformation is complete (Fig. 4c).

Based on the micrographic examination, the amount of DP transformation with aging time at different annealing temperatures was determined. For each aging condition, 5 micrographs were taken at randomly selected areas and the average amount of transformation was calculated.

The Supplementary Figures (Supplementary Figs S2–S4) indicate that the incubation time for discontinuous precipitation decreases on increasing the annealing temperature, up to 650 °C where the DP transformation could still go to completion after aging for 30 min. However, on annealing the alloy at 700 °C, the amount of transformation reaches a maximum of around 67% after 30 min and does not proceed further at this temperature even after aging for 120 min.

### Nanoplates observation

**Figure 1:**
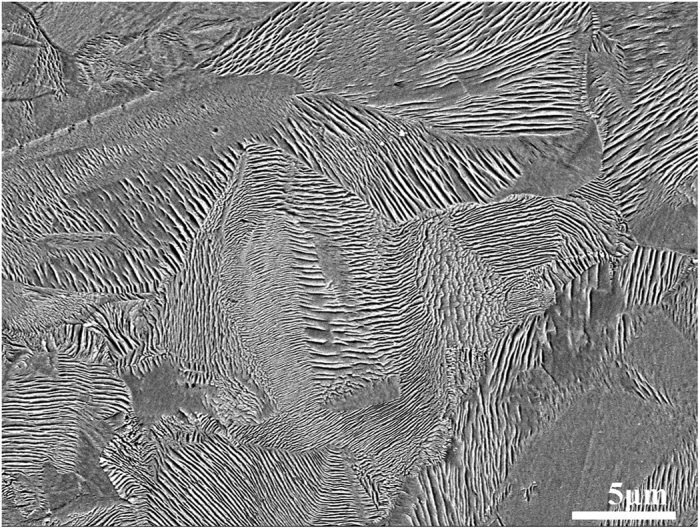
SEM image of etched Ni- 49at.%Co-12at.%Al alloy, 30 min aging at 600 °C: showing nanoplates with different orientations.

**Figure 4:**
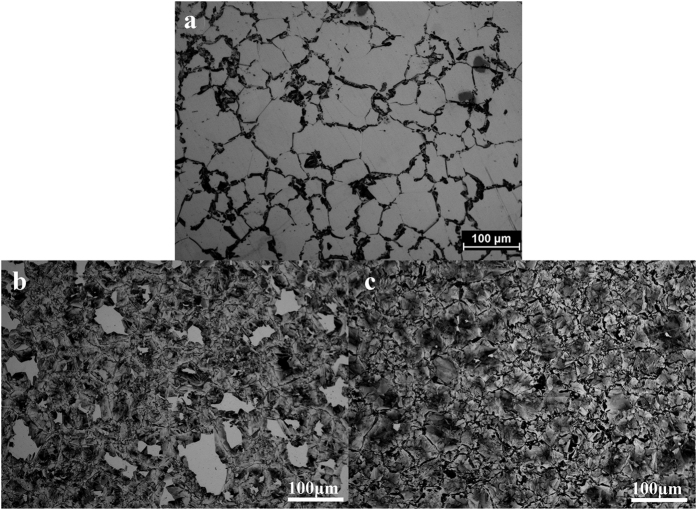
Optical micrographs of Ni 49-12 after aging at 600 °C for different time. The dark area indicates the transformed region. (**a**) After aging for 2 min. (**b**) 5 min. (**c**) 30 min.

After electrolytic etching, the γ phase in the DP transformed region was removed, leaving the plates of γ′ phase. An SEM image of a fully transformed sample is shown in Fig. 1. It can be seen that for an aging time of 30 min, the DP transformation goes to completion and the whole area is transformed to nanoplates with different orientations.

In Supplementary Fig. S5, the microstructure formed at different aging temperatures is presented. This figure shows that the nanoplates exhibit different thickness and spacing according to aging temperature. With an increase of annealing temperature from 550 °C to 650 °C, the average thickness (d) of the plates decreases from 125 nm down to 62.5 nm, while with further increase in the aging temperature to 700 °C, the spacing shows only a slight increase to 67 nm. The change in spacing indicates that the transformation rate of DP increases first and begins to decrease after reaching a maximum, which corresponds to a typical “C-shape” isothermal time temperature-transformation curve. It can also be seen from Supplementary Fig. S5d that many small holes are present in the region untransformed by DP, corresponding to continuous precipitation (CP) particles that have fallen out as the γ matrix was etched. The occurrence of CP decreases the driving force for DP, thereby reducing the velocity of the transformation (with concomitant change in plate spacing) and in some cases preventing the process from going to completion.

### Orientation of the grains

Since a strong {100} grain texture could benefit functionality with {100} oriented plates perpendicular to the foil surface, we investigated methods to produce a strong {100} cube texture. Two different processing methods were investigated: a) thermo-mechanical processing; b) melt spinning.

**Figure 5:**
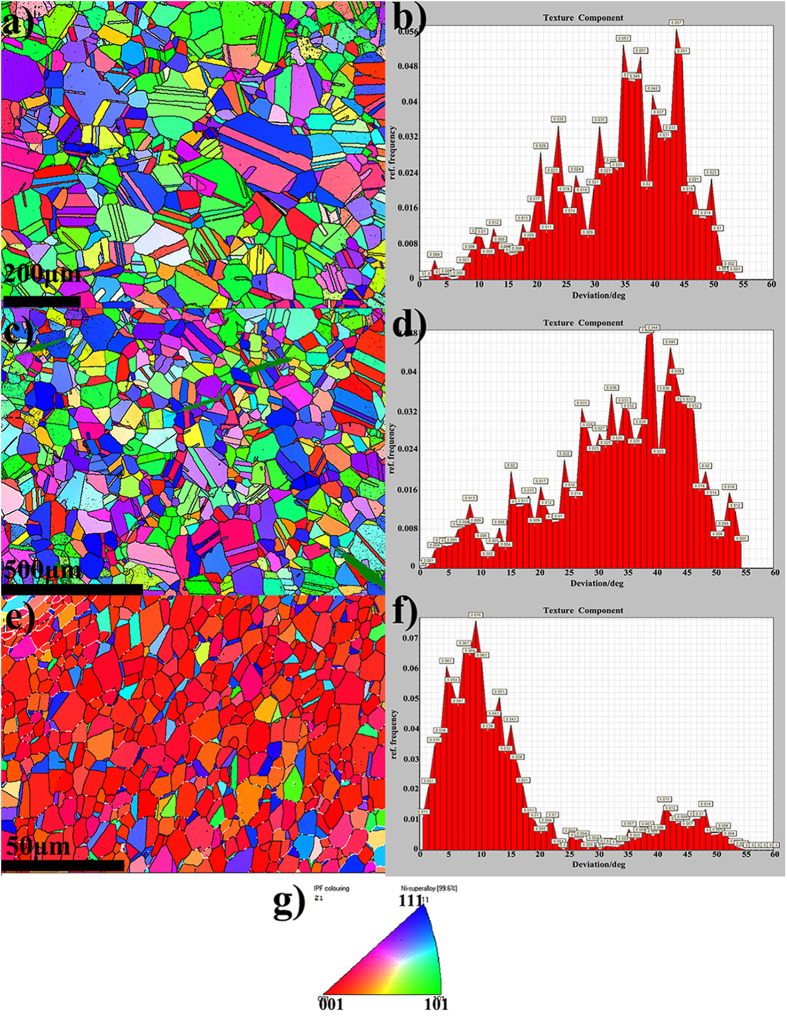
The EBSD results of samples in normal direction (IPFZ) and the texture components. (**a**) Ni49-12 80% cold rolled, heated to 1000 °C in 30 min and held for 1 h followed by quenching. (**b**) Texture component indicates randomly distributed grain orientations. (**c**) Ni49-12 80% cold rolled, heated to 1000 °C in 1 sec and held for 1 h followed by quenching. (**d**) Texture component indicates a similar grain orientation distribution as (**b)**. (**e**) As-cast melt-spun ribbon, Ni49-12, shows a strong <100> texture. (**f**) Texture component indicates a small deviation from <100> around 10°. (**g**) Color code for grain orientations of EBSD pattern.

According to Hutchinson^18^, a strong {100} texture may be produced in cold worked nickel-base alloys by use of a high heating rate to the recrystallization temperature. We therefore heated samples to 1000 °C at two different heating rates in a Gleeble 3500: 0.6 °C/sec and 1000 °C/sec after 80% cold work. The EBSD results are shown in Fig. 5. Each color represents an individual crystal orientation, as illustrated in the legend Fig. 5g. Figure 5a indicates the inverse pole figure (IPF) orientation of the grains from the Z-axis, which is also known as the normal direction (ND). The grain size can be estimated from the micrograph as around 26 μm. Figure 5b shows the texture component with a reference direction of <001> z-axis. The deviation of grain orientation occurs mainly in the 30~50° range. Both the IPFZ and deviation data indicate a close to random distribution. For the second sample (Fig. 5cd) that was heated to 1000 °C within 1 sec, the average grain size was 30 μm. The diffraction results in the IPFZ direction and texture component also indicate an essentially random distribution. The inability to produce a strong cube texture by thermomechanical processing of this material is likely due to the low stacking fault energy in the alloy, which is a result of cobalt addition to nickel^19^.

Since it is known that melt spinning (MS) can produce a strong {100} texture in nickel base alloys^20^, we tried this method and the results are shown in Fig. 5ef. It can be seen that the grain orientation has a strong {100} texture perpendicular to the foil surface. The texture component diagram indicates the high frequency of grains oriented close to <100> with deviations around 10°. This deviation is expected since it is known that in melt spinning the <100> growth direction tends to deviate from the perpendicular with the growth towards the leading edge of the ribbon^21^. The second smaller peak in the range of 42–50 degrees corresponds to the <111> twin orientation relative to the <100> oriented grains. The grain size obtained by MS, which is around 10 μm, is also much smaller than for the thermo-mechanical processing, which is beneficial for enhancing the kinetics of grain boundary nucleated transformations such as DP. The results confirm that a strong {100} cube texture can be achieved by the melt spinning method.

## Discussion

The data on fraction of DP transformation as a function of aging time measured using optical metallography can be analysed based on the JMAK equation^22^,^23^,^24^,^25^:


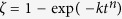


Where *ζ* is the fraction transformed at time *t*; *k* is a rate constant at a given temperature and *n* is the time exponent. If the nucleation rate corresponds to site saturation, which is expected for DP, then *n* = 1. The fitting results for our data are shown in Table 1. It can be seen that the n value in the JMAK equation is less than 1 and decreases with increasing temperature. While *n* = 1 is the ideal value for a site saturated transformation, a DP transformation is often competing with a continuous precipitation transformation in the region ahead of the advancing grain boundary and this reduces the *n* value due to a continuously decreasing driving force^9^.

From the fitting results in Table 1, the isothermal transformation diagram of Ni 49-12 alloy, with an average grain size of 34 μm, was determined and is shown in Supplementary Fig. S6. The curves exhibit the expected ‘C’ curve behavior. For the Ni 49-12 alloy, the nose temperature is 650 °C, at which the discontinuous precipitation begins at the shortest time. This observed nose temperature is consistent with the previously discussed differences in the spacings of the nanoplates in Supplementary Fig. S5. The curves also indicate that the discontinuous precipitation could go to completion at temperatures lower than 650 °C. It only takes 30 min for the alloy to obtain a fully transformed lamellar γ-γ′ microstructure, which is much faster than for the coalescence of discrete γ′ particles reported previously^15^,^16^. At higher temperature, 700 °C, for instance, although the precipitation happens at a fast rate, a complete transformation cannot be achieved, due to the competing kinetics of the continuous precipitation.

**Figure 6:**
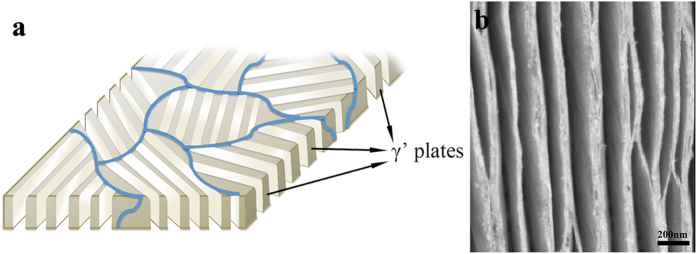
The schematic model and SEM observation of nanoplates microstructure. (**a**) Schematic of foil with strong cube texture-multiple grains with {100} oriented γ′ plates after aging and selective dissolution. (**b**) SEM image of γ′ nanoplates after selective dissolution in Ni49-12 oriented perpendicular to the foil surface.

It is known that for coherent γ/γ′ interfaces in this alloy the orientation relationship is {100}_γ_//{100}_γ′_ and <100>_γ_//<100>_γ′_^9^. In order to maximize the open spacing at a functional surface it is desirable to have a strong cube texture with {100} oriented γ′ plates perpendicular to the surface after aging, as shown schematically in Fig. 6a. If the plate has a strong cube texture and the majority of the grain boundaries are oriented perpendicular to the foil surface prior to aging, such a result can be obtained. The results from EBSD and XRD show that the plates generated via thermo-mechanical processing tend to be randomly oriented with respect to the foil surface. One possible reason is the low stacking fault energy in this alloy due to the high amount of Co^26^. However, using the melt spinning method, in which rapid cooling and directional solidification dominate the grain orientation during growth, a strong {100} texture could be achieved^27^, which can then be further heat treated to produce nanoplates with {100} orientation.

It can be seen from Fig. 6b, that the nanoplates display a favorable microstructure as a potential tunnel and for high surface activity applications such as catalysis. The Brunauer-Emmett-Teller (BET) surface area test results indicate that the surface area increased significantly from 3.2 × 10^−4^ m^2^/g to 8.4 m^2^/g, which demonstrates that this synthesis technique does produce a high surface area that is suitable for catalytic applications. In addition, due to the unique structure and catalytic performance of the Ni_3_Al-alloy plates^28^,^29^, as well as its FCC crystal structure, it is possible for graphene or other kinds of carbon materials to grow on the surface of the nanostructured plates under certain conditions. As a result, there could be a large number of potential applications including: supercapacitors, lithium ion batteries, permeable membranes, heat exchangers, and catalysis^30^,^31^,^32^.

**Figure 7:**
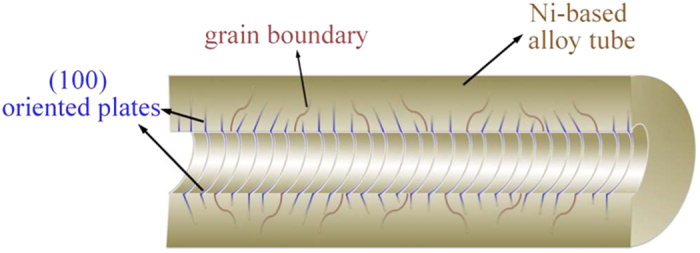
Schematic model of a tube with internal Ni_3_Al nanoplates microstructure.

The nanoscale architecture is controlled through choice of composition and heat treatment, which can control volume fraction and composition of the precipitate and in turn influences the size and spacing of the plates. Mechanical processing can be employed to modify the grain size. The EDS analysis (Table S1.) shows that the average composition of γ′ phase is 26.1 at.%Al, 11.5 at.%Co and 62.4 at.%Ni. One of the advantages of this synthesis technique is that the composition of the nanoplates can be controlled by choice of the original alloy composition and aging temperature, so the value obtained is not unique for the system but can be tuned. Heat treatment times are on the order of an hour and production quantities could be in metric tons utilizing existing metal production infrastructure, making this process commercially viable. As the initial form is a solid solution before precipitation, complex shapes may be produced prior to transformation of the microstructure. For example, for the Ni49-12 alloy used in this work large amounts of cold deformation (up to 95% cold rolling reduction) can be achieved without cracking. For alloys less ductile at room temperature, hot deformation can be performed in the solid solution temperature range. Processing can be modified depending on the application. For example, etching only one side of a plate results in a nanoscale plate structure fixed to a strong, impermeable substrate. Such a structure may find application in tubes where the internal surface has been etched providing a catalytic surface for a fluid flowing in the tube or as a heat exchanger/insulator depending on the fluid. The efficiency of Ni_3_Al as a catalyst for methanol decomposition has been demonstrated previously^28^,^29^. Using a tube with an inner surface that has been processed to produce Ni_3_Al plates as proposed here, shown schematically in Fig. 7, could provide a self-supporting catalyst for methanol decomposition. The unetched but precipitated structure would serve as a strong support for the functional material and is structurally sound at the temperatures needed for the process. Etching both sides of a thin foil can give a permeable membrane, the tortuosity of the structure maintaining integrity. This synthesis technique is generally applicable to any alloy system undergoing a complete discontinuous precipitation transformation by a coupled growth mechanism and so it may be applicable in a wide range of applications. For example, Ag nanoplate structures which are of use in anti-bacterial applications could be produced if a suitable alloy system fulfilling the criteria is found. Similarly Ti nanoplates could be produced which can be functionalized by *in-situ* conversion to TiO_2_, or Mo nanoplates converted to MoS_2_ for battery applications^33^. The nanoplate structures may even find application as structural materials similar to metallic foams^34^ or as low friction surfaces for wear applications. If the nanoplates have magnetic or shape memory properties then it may be possible to design large scale mechanical devices based on these functionalities.

The major challenge to the application of this technique for a specific nanoplate material is in finding a suitable alloy system that can meet the criteria of complete DP and selective removal of one of the phases. It is not sufficient that there be a high temperature single phase that on cooling transforms to a two phase structure since we need that transformation to be by DP. Since our understanding of the competitive kinetics of DP and CP is incomplete it is not possible to know *a priori* the occurrence of DP let alone if it can go to completion. This is a neglected area of phase transformation study that needs to be investigated since it can have important applications as discussed in this work.

## Conclusions

We have shown that, self assembled, aligned nanoplates can be synthesized by a novel method involving the discontinuous precipitation of a γ′ phase followed by selective dissolution of the matrix in a Ni-49Co-12Al alloy. The precipitation behavior was studied in the alloy after cold work and thermal processing as well as texture analysis. The following conclusions may be made:

1. Aligned nanoplate structures can be produced by selectively etching a DP matrix. This structure is self-supporting due to the tortuosity and branching of the precipitate phase. This synthesis method has been demonstrated using a Ni-49at.%Co-12at.%Al alloy.
2. A complete discontinuous transformation in the Ni 49-12 alloy can be achieved in times as short as 30 min. Thus, the large scale production of such structures is feasible using conventional heat treatment facilities.
3. The discontinuous precipitation kinetics exhibits a typical “C” shape transformation curve with a nose temperature of about 650 °C. Through control of the aging parameters and alloy composition, it is possible to produce plates of different thickness and different spacings.
4. The thermo-mechanical processing paths used in this work failed to produce a strong {100} texture. A melt spinning technique was successful in producing a strong {100} cube texture in a thin foil.
5. The synthesis technique is generally applicable to any alloy system in which DP goes to completion and one phase can be selectively removed. The nanoplates are self-assembled, self-supported and well aligned if the precipitate is coherent with the matrix.

## Methods

### Preparation of Ni-Co-Al alloy samples

The alloy composition selected in this work was Ni49-12. This alloy has been shown to undergo a complete discontinuous precipitation transformation over a range of aging temperatures^9^. The material was arc melted from elemental starting materials of Ni shot, Co pieces and Al shot (all materials with 99.99% purity) into ingots weighing from 60 to 70 grams. The ingots were flipped and remelted 3 times. The ingots were then homogenized under an argon atmosphere at 1000 °C for 24 hrs. After homogenization, the material was cold-rolled 90%, resulting in a 1 mm thick foil. The foil was then solution treated at 1000 °C for 60 minutes and quenched to produce a single-phase γ structure followed by aging for different times at various temperatures ranging from 550 °C to 700 °C. A free-jet melt spinning method was used to produce thin Ni-Co-Al foils, in which the molten alloy was ejected onto a water cooled copper wheel rotating with a surface speed of 31 m/s, resulting in 50 μm thick foils.

### Selective etching for analyzing DP transformation

The material was examined optically after short time electrolytic etching in a solution of 2% ammonium sulphate and 2% citric acid in water and the fraction of discontinuous γ-γ′ precipitation was determined by image analysis. This etchant does not reveal grain boundaries unless there is some DP present on the boundary. To establish the grain size after solution treatment the samples were etched with Marble’s reagent. The aged material was then further electrolytically etched in the same solution for 24 hours. This selectively removes the γ matrix phase from the structure leaving behind the γ′ plates.

### Nanoplates characterization

A Scanning Electron Microscope (SEM) was used to observe the nanoplate structures produced via the DP process. In order to investigate the influence of processing on texture formation in the Ni 49-12 alloys, thermo-mechanical processing (using a Gleeble 3500) and melt spinning methods were employed in this work. Electron Backscattered Diffraction (EBSD) analysis was performed to quantify the texture development in these samples. BET analysis was used to measure the surface area of the nanoplates for potential catalyst applications.

## Additional Information

**How to cite this article**: Zhou, Y. *et al*. The Large Scale Synthesis of Aligned Plate Nanostructures. *Sci. Rep.* **6**, 29972; doi: 10.1038/srep29972 (2016).

## Supplementary Material

Supplementary Information

## Figures and Tables

**Figure 2:**
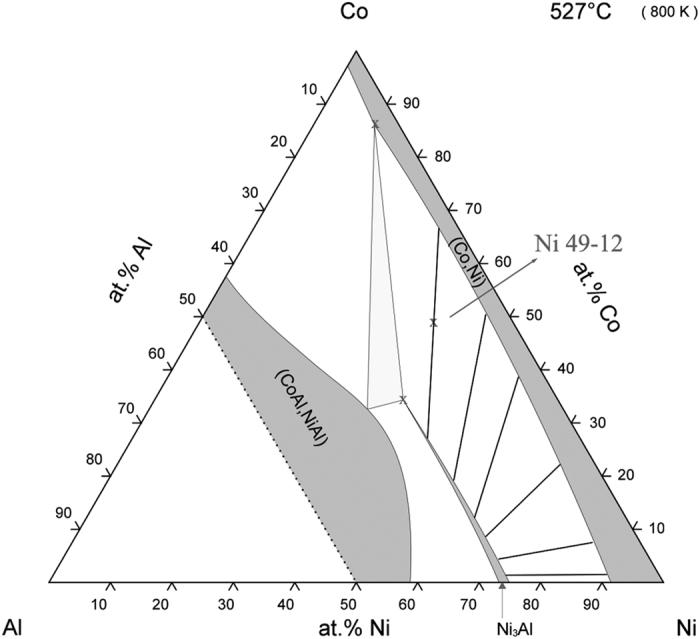
Ni-Co-Al isothermal section at 527 °C showing schematically the rotation of the tie line for γ + γ′ equilibrium with increasing cobalt content^14^.

**Figure 3:**
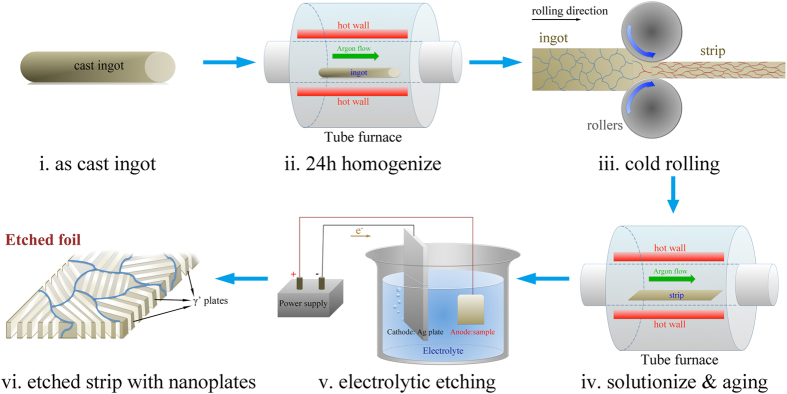
Process to produce nanostructured plates. The material was prepared by arc melting from elemental starting materials. The ingots were then homogenized at 1000 °C in the furnace with Ar protection, quenched in water and subsequently cold rolled 90%, resulting in a 1 mm thick foil. This process removes the as-cast dendritic structure and chemical segregation resulting from solidification. The foil was then solution treated at 1000 °C and quenched to produce a single-phase γ structure followed by aging for different times at various temperatures ranging from 550 °C to 750 °C. Finally, to obtain only the nanoplate precipitate microstructure, we used a selective etching method to remove the γ phase. The electrolytic etchant used is 2% Ammonium Sulphate +2% Citric Acid solution in water. The applied current during the dissolution process was 0.5A and the etching time was 24 hours.

**Table 1:** Fitting results using JMAK kinetics equation.

Temperature (°C)	*k*	*n*	Time (s)		
			5%	50%	95%
550	0.0059	0.89	11.4	214.2	1114
600	0.0112	0.83	6.2	143.8	838
650	0.0347	0.65	1.8	100.3	953
700	0.0955	0.31	2.2	188.3	2299
