# Supplementary material for: The Large Scale Synthesis of Aligned Plate Nanostructures
Source: Sci Rep. 2016 Jul 21;6:29972. doi: 10.1038/srep29972 (PMC4954993; doi:10.1038/srep29972)
Supplement: Supplementary Information [file srep29972-s1.pdf]

The Large Scale Synthesis of Aligned Plate Nanostructures

Yang Zhou[1], Philip Nash[1*], Tian Liu[1], Naiqin Zhao[2], Shengli Zhu[2]

1. Thermal Processing Technology Center, Illinois Institute of Technology, Chicago, IL 60616, USA
2. Key Laboratory of Composite and Functional Materials, School of Materials Engineering, Tianjin University, Tianjin, 300072, China

*Corresponding author: Professor Philip Nash, nash@iit.edu

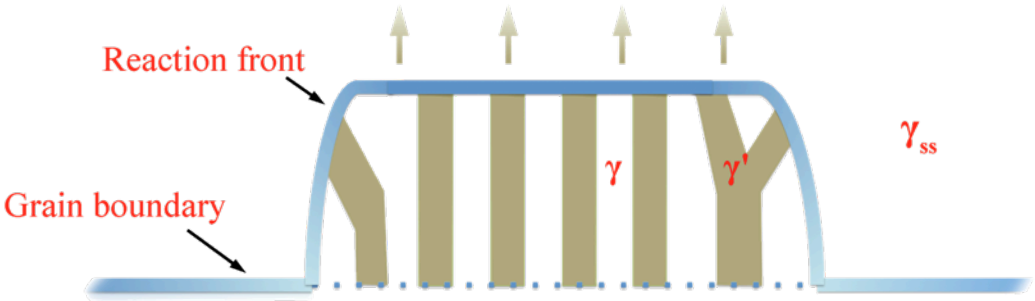

Supplementary Figure S1. Schematic model of discontinuous precipitation: also known as cellular precipitation, is a transformation which involves the cellular growth of alternating layers of the precipitate phase and matrix phase behind a moving high-angle grain boundary.

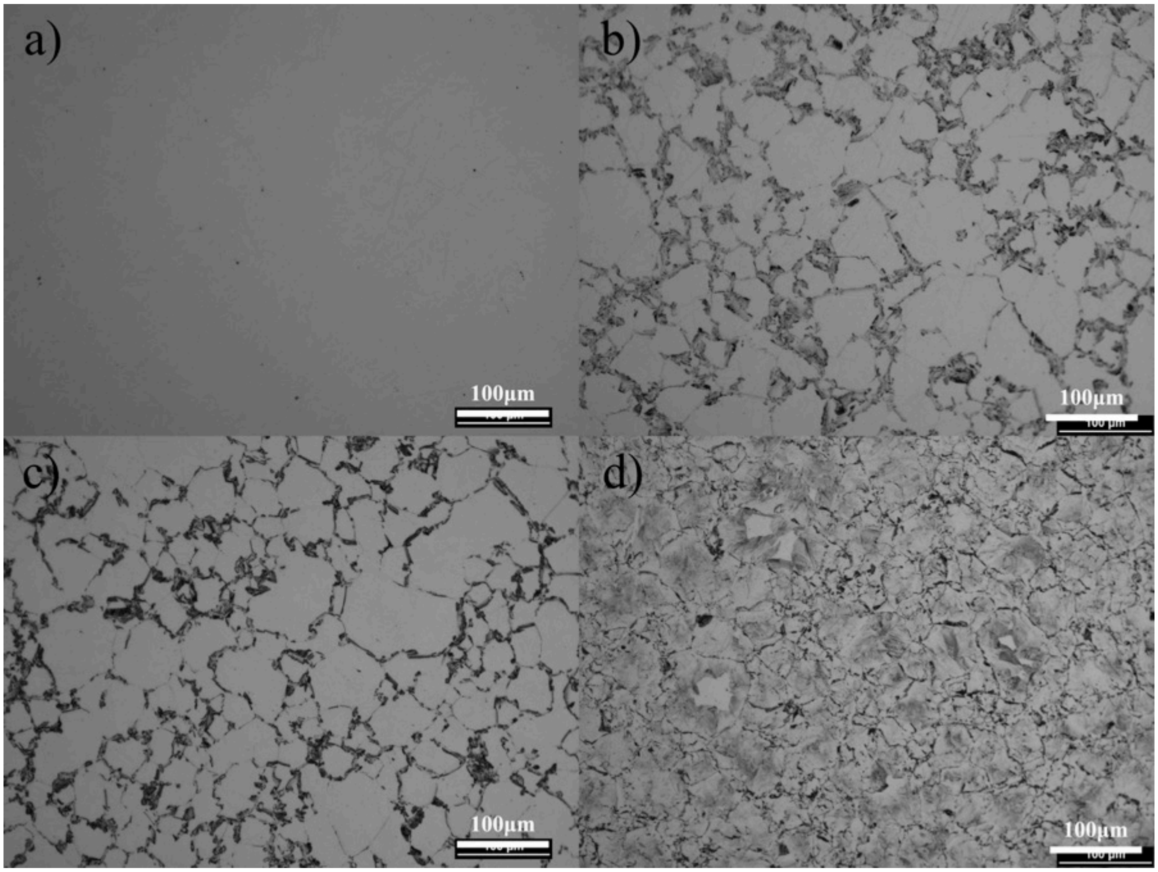

Supplementary Figure S2. The metallograph of Ni 49-12 after aging at 550℃ for a) 45sec; b) 1min; c) 3min; d) 30min.

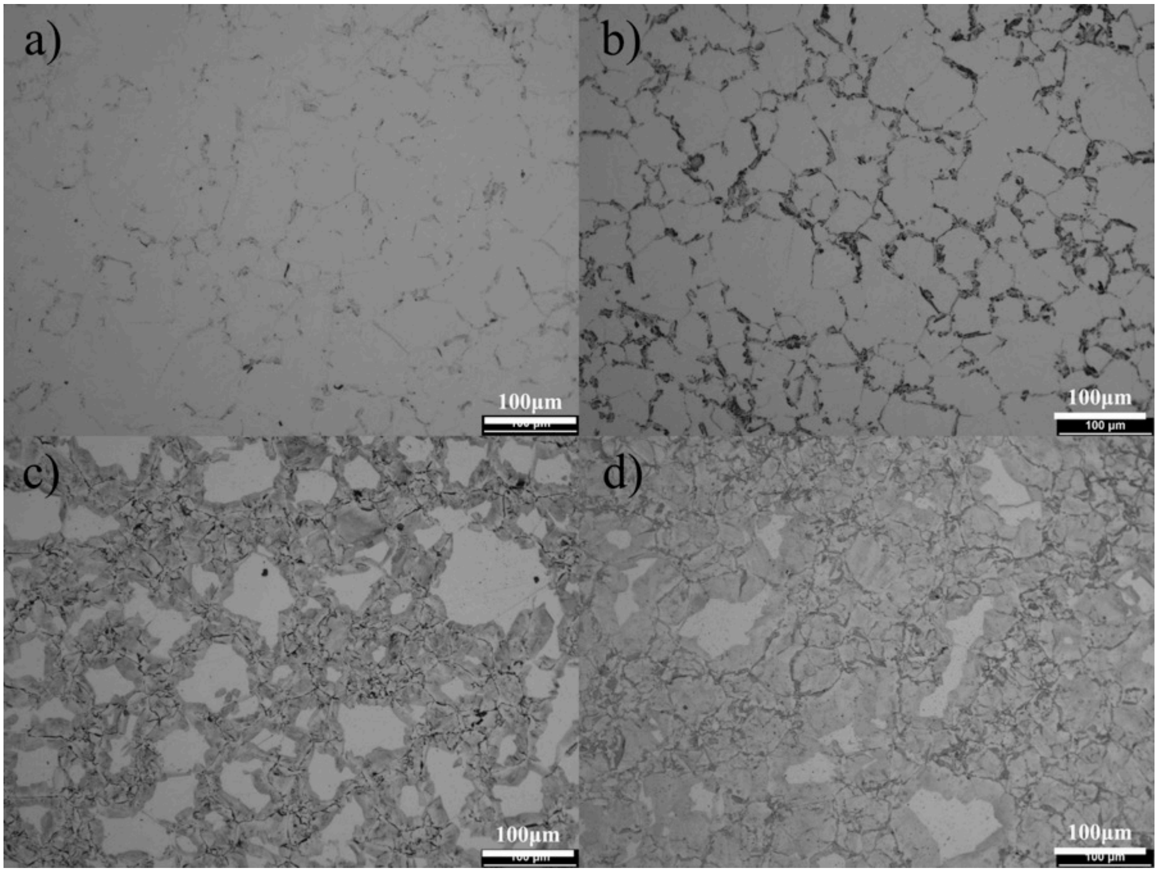

Supplementary Figure S3. The metallograph of Ni 49-12 after aging at 650℃ for a) 45sec; b) 1min; c) 5min; d) 30min.

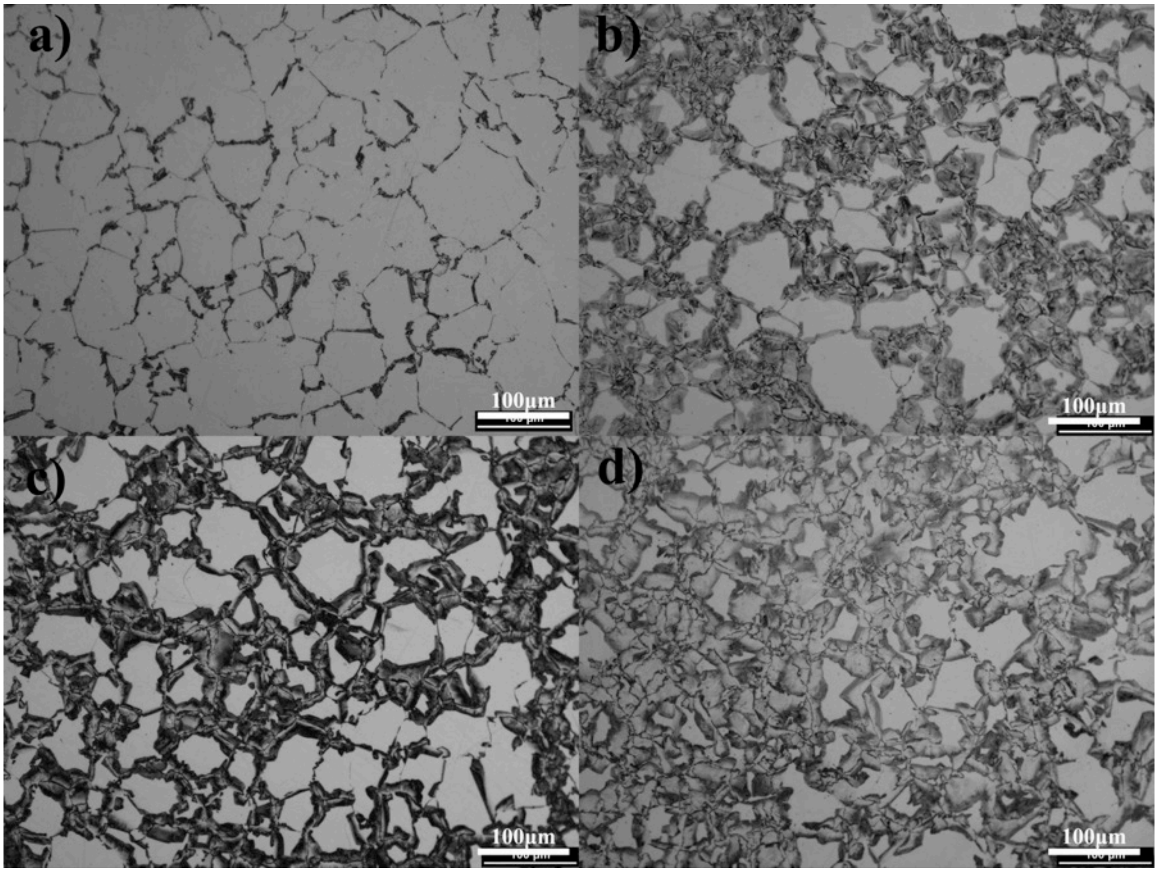

Supplementary Figure S4. The metallograph of Ni 49-12 after aging at 700℃ for a) 1min; b) 5min; c) 30min; d) 120min.

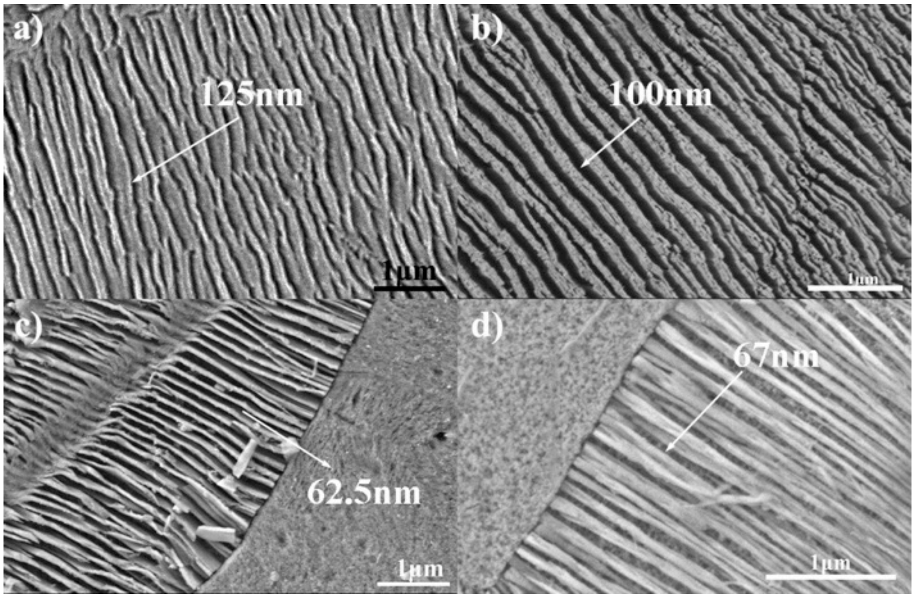

Supplementary Figure S5. SEM images of the nano structure of Ni 49-12 alloy after 30min aging at a) 550℃; b) 600℃; c) 650℃; d) 700℃.

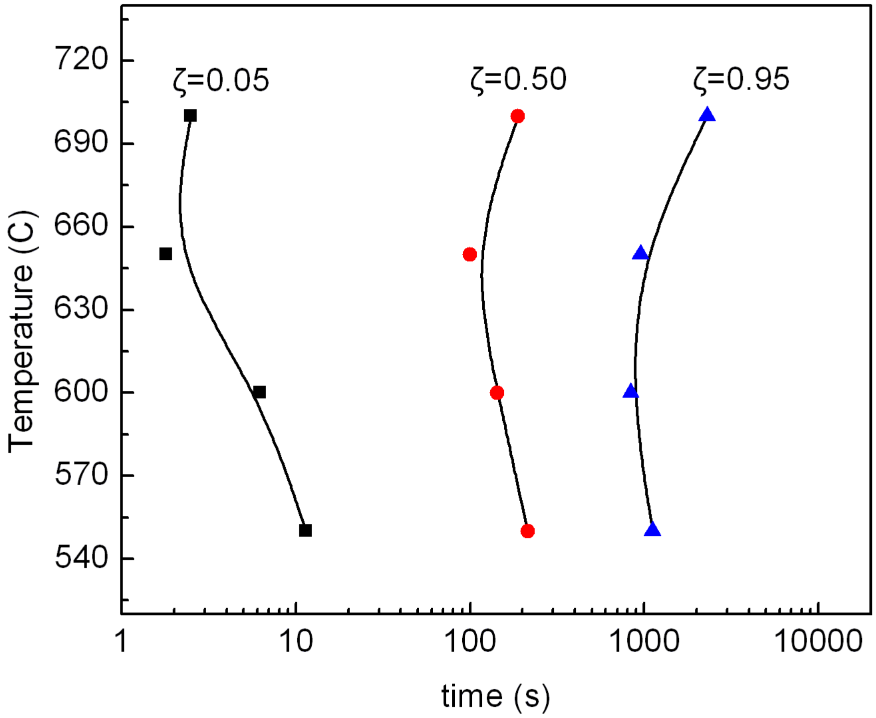

Supplementary Figure S6. TTT diagram of Ni 49-12 with an average grain size of 34μm.

Table S1. The composition of γ' phase from EDS analysis

Spectrum	Al/at.%	Co/at.%	Ni/at.%
Spectrum 1	27.4	11.6	61.0
Spectrum 2	28.02	11.13	60.85
Spectrum 3	24.10	9.78	66.12
Spectrum 4	23.22	12.81	63.97
Spectrum 5	27.94	12.06	60.00
Mean.	26.1	11.5	62.4
Std. Deviation	2.3	1.1	2.6
